# Astrocytes Protect Neurons in the Hippocampal CA3 Against Ischemia by Suppressing the Intracellular Ca^2+^ Overload

**DOI:** 10.3389/fncel.2018.00280

**Published:** 2018-08-24

**Authors:** Chuanqi Sun, Yasuko Fukushi, Yong Wang, Seiji Yamamoto

**Affiliations:** ^1^Department of Innovative Medical Photonics, Preeminent Medical Photonics Education and Research Center, Hamamatsu University School of Medicine, Hamamatsu, Japan; ^2^Department of Neurosurgery, First Affiliated Hospital of China Medical University, Shenyang, China

**Keywords:** astrocyte, brain ischemia, neuroprotection, intracellular Ca^2+^ overload, delayed neuronal death

## Abstract

In the hippocampus, delayed neuronal death is normally seen in neurons of the CA1 region but not in those of the CA3 region. Astrocytes have been reported to play multiple supporting or pathological roles in neuronal functioning. While evidence indicates that astrocytes could exert neuroprotective effects following ischemia, the possible underlying mechanisms remain unclear. We aimed to investigate the roles of astrocytes in the process of delayed neuronal death following transient forebrain ischemia. L-α-aminoadipic acid (L-α-AAA), an astrocyte-selective gliotoxin, was injected into the hippocampal CA3 region of rats through a cranial window to selectively damage astrocytes. Immunofluorescence staining of glial fibrillary acidic protein (GFAP) was used to evaluate the effect of L-α-AAA on astrocyte numbers. Three days after the L-α-AAA injection, transient forebrain ischemia was induced by a modification of the four-vessel occlusion procedure. Seven days after transient forebrain ischemia, hematoxylin-eosin staining was performed to reveal the morphology of hippocampal pyramidal neurons. In rats with ischemia and reperfusion, regional cerebral blood flow (rCBF) and change in intracellular Ca^2+^ concentration ([Ca^2+^]_i_) were separately measured in CA1 and CA3 regions. L-α-AAA injection significantly decreased the number of astrocytes in CA3, but did not affect the pattern of rCBF changes upon ischemia/reperfusion. Seven days after transient forebrain ischemia, in rats receiving L-α-AAA, delayed neuronal death comparable with that in CA1 was observed in the CA3 region. In addition, the pattern of increase in [Ca^2+^]_i_ due to transient forebrain ischemia was completely changed in the hippocampal CA3. The loss of astrocytes induced a persistent increase in [Ca^2+^]_i_ in the CA3 region following transient ischemia, similar to what is observed in the CA1 region. Our study indicates that astrocytes in the hippocampal CA3 region exert neuroprotective effects following transient forebrain ischemia and act by suppressing the intracellular Ca^2+^ overload. Furthermore, our study will most likely provide a new therapeutic strategy for brain ischemic diseases, targeted to astrocytes.

## Introduction

In past decades, astrocytes have generally been considered as a supportive glial cell in the central nervous system (CNS). However, in recent years, studies on astrocytes have shown that they play multiple roles in neuronal functioning, including both physiological and pathological processes (Sofroniew and Vinters, 2010; Clarke and Barres, 2013). Astrocytes have extensive contacts with neuronal synapses, axons, blood vessels, and even other adjacent glial cells, and participate in the regulation of information processing and synaptic transmission by expressing various neurotransmitters and receptors (Nedergaard et al., 2003; Halassa et al., 2007; Perea et al., 2009; Sofroniew and Vinters, 2010). Astrocytes can regulate the release of active synaptic molecules such as glutamate, GABA, D-serine, ATP, and adenosine, and affect neurons directly and interactively in the process of regulating synaptic functions (Rossi et al., 2007; Sofroniew and Vinters, 2010). Moreover, astrocytes participate in the regulation of fluid balance, ionic and pH homeostasis, CNS metabolism, and even the integrity of the blood-brain barrier (Sofroniew and Vinters, 2010). In recent years, accumulating evidence has shown that astrocytes play an essential role in ischemic stroke. Most of these studies indicate that astrocytes exert neuroprotective effects through various mechanisms. Astrocytes can protect neurons from insults due to oxidative stress and excitotoxicity after brain ischemia (Rossi et al., 2007; Takano et al., 2009; Zhao and Rempe, 2010). A 2007 review showed that during brain ischemia, astrocytes may provide glycogen stores to support neurons and regulate the release of transmitters critical in ischemic brain damage, including glutamate, D-serine, and adenosine (Rossi et al., 2007). After brain ischemia, astrocytes were reported to protect neurons by increasing neuronal glutathione levels (a response to oxidative stress), releasing protective factors such as erythropoietin, and regulating ionic homeostasis (Annunziato et al., 2013).

Ischemic stroke can lead to neuronal death or brain damage through various mechanisms, including glutamate and Ca^2+^ toxicity, oxidative stress, mitochondrial dysfunction, acidosis, and inflammation (Szydlowska and Tymianski, 2010; Ding, 2014; Mayor and Tymianski, 2017). Deprivation of the oxygen and energy supply after ischemic stroke induces loss of ionic gradients and membrane depolarization in neurons. Following this, excitatory neurotransmitters (e.g., glutamate) are released and accumulate in the extracellular space and over-stimulate the neuronal receptors, which finally cause a Ca^2+^ influx into neurons (Deb et al., 2010; Szydlowska and Tymianski, 2010). The high [Ca^2+^]_i_ can also arise from a malfunction of internal storage of calcium in organelles such as the endoplasmic reticulum and mitochondria (Bodalia et al., 2013). The intracellular Ca^2+^ overload in neurons is critical to the process of neuronal death because it triggers numerous downstream effectors and contributes to the degradation of membranes and proteins (Szydlowska and Tymianski, 2010; Bodalia et al., 2013). Astrocytes may regulate neuronal [Ca^2+^]_i_ by taking up or releasing excitatory neurotransmitters such as glutamate, ATP, and adenosine (Rossi et al., 2007). Moreover, astrocytes express ionic transporters, such as Na^+^-K^+^-ATPase, Na^+^-Ca^2+^ exchanger, and Na^+^-K^+^-Cl^-^ cotransporter, which may help maintain Ca^2+^ homeostasis after brain ischemia (Rossi et al., 2007; Ding, 2014).

Astrocytes may thus protect neurons against ischemia. However, the mechanisms of such protection remain controversial and unclear. Considering the known, multiple effects of astrocytes on neurons and their ability to regulate Ca^2+^ homeostasis after brain ischemia, we tested *in situ* our hypothesis that astrocytes may protect neurons against ischemic injury by suppressing the step of post-ischemic overloading of neurons with intracellular Ca^2+^.

## Materials and Methods

### Animals and Treatments

**FIGURE 1:**
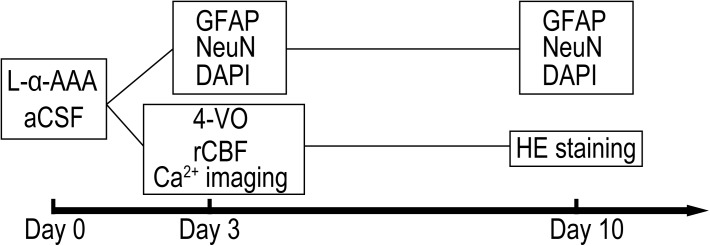
Shown is the timeline of the experiment. Abbreviations are as follows: “L-α-AAA” or “aCSF,” injection of L-α-aminoadipic acid or artificial cerebrospinal fluid; “GFAP” or “NeuN,” immunofluorescence staining of glial fibrillary acidic protein (GFAP) or neuronal nuclear; “DAPI,” DAPI (4′,6-diamidino-2-phenylindole) staining; “4-VO,” administration of 4-vessel-occlusion transient forebrain ischemia; “rCBF” and “Ca^2+^ imaging,” regional cerebral blood flow measurement and *in situ* Ca^2+^ fluorescence imaging are performed during brain ischemia/reperfusion; “HE staining,” hematoxylin-eosin staining of fixed brain slices for cell counting.

Male, adult Sprague-Dawley rats (Japan SLC, Inc., Hamamatsu, Japan) weighing 280–320 g were used. Animals were housed at the Animal Center at Hamamatsu University School of Medicine (HUSM) under a 12:12-h light/dark cycle at a constant temperature of 25°C with free access to food and water. All experiments were performed according to the guidelines for the care and use of animals established by the Physiological Society of Japan. The experimental protocol was approved by the Committee on Ethics in Animal Experimentation at HUSM. Experiments were randomized and blinded and powered to detect effect sizes at 80%. Rats were anesthetized with 4% halothane, remainder O_2_, for induction and 1–2% isoflurane, remainder O_2_, for maintenance. Rectal temperature was measured and maintained at 37 ± 0.5°C throughout the experiment by a thermostatically controlled heating pad. The experimental schedule is shown in **Figure 1** as a time sequence. Gliotoxin (L-α-aminoadipic acid, L-α-AAA) or artificial cerebrospinal fluid (aCSF) was injected into the hippocampal CA3 region at day 0. Glial fibrillary acidic protein (GFAP), neuronal nuclear (NeuN) and 4′,6-diamidino-2-phenylindole (DAPI) staining were performed at days 3, and 10. Four-vessel-occlusion, transient forebrain ischemia was administered at day 3, with simultaneous real-time, *in situ* measurement of regional cerebral blood flow (rCBF) and intracellular Ca^2+^. To confirm delayed neuronal death, brain slices were stained with hematoxylin and eosin (HE) staining 7 days after transient forebrain ischemia (day 10).

### Gliotoxin Injection

Rats were anesthetized and placed on a stereotaxic apparatus (Narishige Co., Ltd., Tokyo, Japan). An astrocyte-selective gliotoxin, L-α-AAA was applied. L-α-AAA could selectively damage astrocytes but does not damage the other cells such as neurons, microglia, oligodendroglia, and endothelial cells (Takada and Hattori, 1986; Khurgel et al., 1996). A glass micropipette filled with a solution L-α-AAA (0.1 mol/L in aCSF, A7275, Sigma) was inserted into the right hippocampal CA3 through a 3 mm× 3 mm cranial window. Insertion coordinates were 3.6 mm caudal and 3.5 mm lateral to the bregma and 3.2 mm deep to the dura; angle, 90°. A pressure pulse of 0.1 MPa was applied to the pipette using an IM-3 microinjector (Narishige Co., Ltd., Tokyo, Japan) to eject 10 μL of solution. In the sham-treated group, 10 μL of aCSF were injected into the same area using the same method. After the injection, anesthesia was discontinued and the rats were returned to the Animal Center for subsequent procedures.

### Immunofluorescence Staining

See **Figure 1** for timing. The immunohistochemistry procedure for fluorescence staining of GFAP has been reported extensively. Rats were deeply anesthetized and then perfused transcardially with 4% paraformaldehyde in phosphate-buffered saline (PBS) at 0.1 mmol/L, pH 7.4 (PFA). The brains were then removed and stored overnight in PFA. Brain tissues were dehydrated in sucrose solution (10% followed by 30%), then frozen in embedding medium with liquid nitrogen. Coronal frozen sections 20 μm thick were then collected for immunofluorescence staining. Brain slices were rinsed in 0.2% Triton X-100 solution (in 0.1 mmol/L PBS, pH 7.4, 10 min × 3) and then incubated with background blocking buffer (normal goat serum, ab7481, Abcam Co., Tokyo, Japan) for 1 h at room temperature. The brain slices were then incubated with rabbit anti-GFAP (1:5,000, ab7260, Abcam) and mouse anti-NeuN (1:500, ab104224, Abcam) overnight at 4°C. Then the slices were rinsed in blocking buffer and incubated with fluorescent secondary antibody, Alexa Fluor 488 goat anti-rabbit IgG (1:1,000, ab150077, Abcam) and Alexa Fluor 555 goat anti-mouse IgG (1:1,000, ab150118, Abcam), for 1 h at room temperature (20–25°C). Slices were rinsed again in PBS following secondary-antibody incubation and then mounted for fluorescence microscopy using an antifade mounting medium with DAPI (ProLong^TM^ Diamond, Thermo Fisher Scientific Co., Tokyo, Japan). Fluorescence images were collected using NanoZoomer 2.0-HT (Hamamatsu Photonics Co., Ltd., Hamamatsu, Japan). Numbers of GFAP&DAPI-positive (GFAP&DAPI ^+^) cells were calculated in a 0.08 mm^2^ area (up to 100 μm above and below the CA3 nerve cell layer, 400 μm width to the left and right) using NDP.view 2.0 software (Hamamatsu Photonics Co., Ltd., Hamamatsu, Japan).

### 4-Vessel-Occlusion, Transient Forebrain Ischemia Model

Transient forebrain ischemia was administered 3 days after L-α-AAA injection according to previously published methods (Pulsinelli and Brierley, 1979; Pulsinelli and Buchan, 1988), with some modification (Wang et al., 2010). Under anesthesia, an incision 1.5 cm in length was made behind the occipital bone directly overlying the first two cervical vertebrae. The paraspinal muscles were separated from the midline. Bilateral vertebral arteries were completely occluded through the alar foramina of the first cervical vertebra by cauterization. The incision was then closed with a surgical suture. The bilateral common carotid arteries were then exposed via a ventral midline incision and a balloon vessel-occluder (VO-2; Unique Medical Co., Ltd., Tokyo, Japan) was placed loosely around each artery. By tightening and releasing the balloons, we induced an ischemic period 10 min in duration followed by reperfusion (Wang et al., 2010). Use of the balloon occluders facilitated the implementation of real-time, *in situ* rCBF measurements and Ca^2+^ fluorescence imaging before, during, and after the ischemic event.

### Regional Cerebral Blood Flow (rCBF) Measurement

The rCBF measurements in the CA1 and CA3 regions were by laser-doppler flowmetry (LDF) implemented with the ALF2100 flowmeter (Advance, Tokyo, Japan). Similar to the procedure for Ca^2+^ fluorescence imaging, the laser-Doppler probe (needle probe type, diameter 0.5 mm) was stereotactically inserted to the CA1 or CA3 regions through the cranial window. Data were recorded every 2 min from 10 min before transient forebrain ischemia to 20 min after reperfusion (40 min in total).

### *In situ* Ca^2+^ Fluorescence Imaging

**FIGURE 2:**
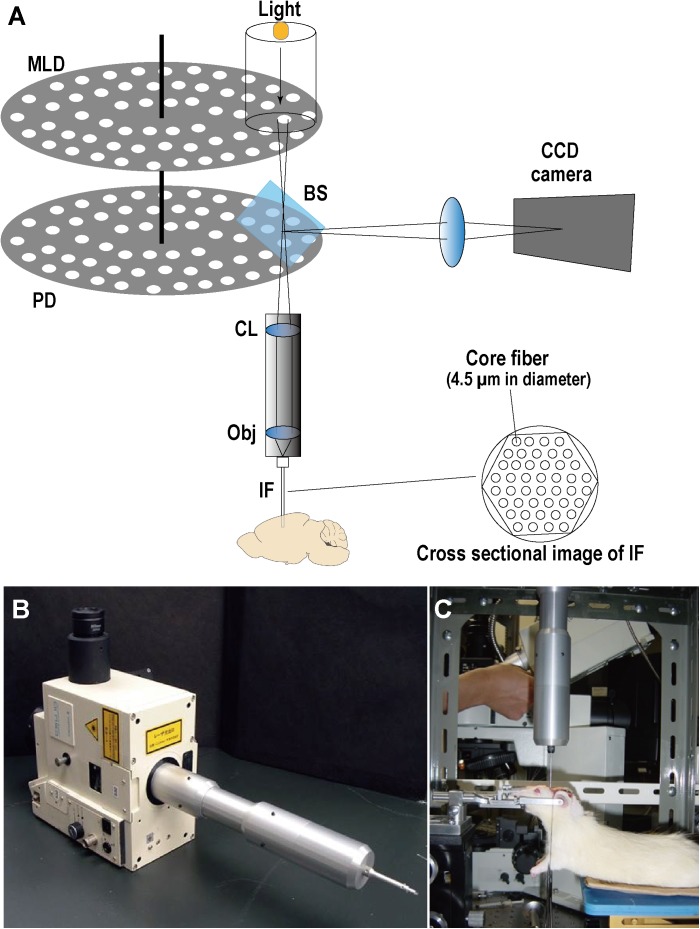
Optical schematic of the fiber-coupled confocal microscope (FCM) system for real-time intravital imaging. **(A)** Illustration of FCM components. MLD, microlens array disk; PD, pinhole array disk; BS, beam splitter; CL, collimator lens; Obj, objective lens; IF, imaging fibers (i.e., a fiber bundle); CCD, charge-coupled device. **(B)** Image of the multi-pinhole confocal scanner unit (CSU-21, Yokogawa, Japan) coupled with the imaging fiber bundle. **(C)** Real-time Ca^2+^ fluorescence imaging in CA1/CA3 *in situ* with FCM.

To investigate the intracellular Ca^2+^ response to transient forebrain ischemia in hippocampal CA1 and CA3 regions, Ca^2+^ fluorescence imaging was performed *in situ* by a fiber-coupled confocal microscope (FCM), which used imaging fiber-optics coupled to a multi-pinhole confocal scanner unit (CSU-21; Yokogawa, Tokyo, Japan) (**Figure 2**). The FCM system allowed observation of the *in situ* Ca^2+^ fluorescence image deep inside the brain (Sakurai et al., 2006). Three days after L-α-AAA or aCSF injection, rats were fixed in a stereotactic frame under anesthesia. A new cranial window 3 mm × 3 mm square was made for accessing CA1, centered at 3.6 mm caudal and 2 mm lateral to the bregma. The previous cranial window for CA3 (3.6 mm caudal and 3.5 mm lateral to the bregma) was reopened. The dura was then opened carefully without damage to the cortical surface. The Ca^2+^ indicator, fluo3/AM (Dojindo Co., Ltd., Tokyo, Japan), was applied by the pressurized-bolus injection method (Wang et al., 2010). A glass micropipette filled with fluo3/AM solution (2.5 μL, 440 μmol/L) was inserted slowly into the CA1 region (angle, 90°; 2.0 mm below the dura) or the CA3 region (angle, 90°; 3.2 mm below the dura). A pressure pulse of 0.1 MPa (IM-3, Narishige Co., Ltd., Tokyo, Japan) was applied to inject the dye solution. Rats were stabilized for 30 min after injection. An imaging fiber coupled to the laser confocal microscope was then inserted into the CA1 or CA3 region to observe the Ca^2+^ fluorescence. Rats were stabilized for a further 5 min, and then fluorescence images were recorded every 30 s beginning 5 min before transient forebrain ischemia and continuing until 20 min after reperfusion (35 min in total).

### Imaging of Fluoromicrospheres in Hippocampus During Ischemia and Reperfusion

The 4-vessel-occlusion procedure leads to a decrease in the hemoglobin content of the brain. Because hemoglobin absorbs both the excitation and emission light, the occlusion process may affect the fluorescence intensity recorded from the calcium-sensitive dye inside the brain. To control for the artefactual effects of ischemia on apparent fluorescence intensity, fluoromicrospheres 0.2 μm diameter (*E*x/*E*m: 490/510 nm), which had excitation and emission properties similar to those of fluo3/AM (*E*x/*E*m: 508/527 nm), were injected into the hippocampal CA3 region. The fluorescence of fluoromicrospheres is not biologically affected by ischemia/reperfusion. Fluorescence images were taken before and after a 10 min period of forebrain ischemia in the same manner as for fluorescence imaging with fluo3/AM. In Ca^2+^ fluorescence imaging, the data collected during ischemia/reperfusion were corrected based on the results obtained with fluoromicrospheres. The corrected data were calculated using the experimental equation *P*_corrected_ = *P*_dye raw_/*P*_fluoromicrospheres_ %, where *P*_corrected_ is the corrected percentage of fluorescence intensity of fluo3/AM; *P*_dye raw_ is the percentage of the fluorescence intensity of fluo3/AM; *P*_fluoromicrospheres_ is the percentage of the fluorescence intensity of fluoromicrospheres.

### HE Staining

The method of HE staining has been widely reported and described. After staining, the number of pyramidal neurons in the CA1 and CA3 regions of the control, L-α-AAA, and aCSF-treated groups were calculated, each measurement based on a 500 μm length of the somatic layer, which appears as a line in coronal cross section. The control rats were the same individuals as the L-α-AAA group, but using the contralateral, un-injected side. Image J software was used.

### Statistical Analysis

Data were expressed as mean ± SD or medians and percentiles depending on their distribution. Group differences were analyzed using Mann–Whitney *U*-test to compare between two groups, and Kruskal–Wallis test for multiple comparisons. Differences were considered significant at *p* < 0.05.

## Results

### L-α-AAA Injection Induced Astrocytic Depletion in the Hippocampal CA3 Region

**FIGURE 3:**
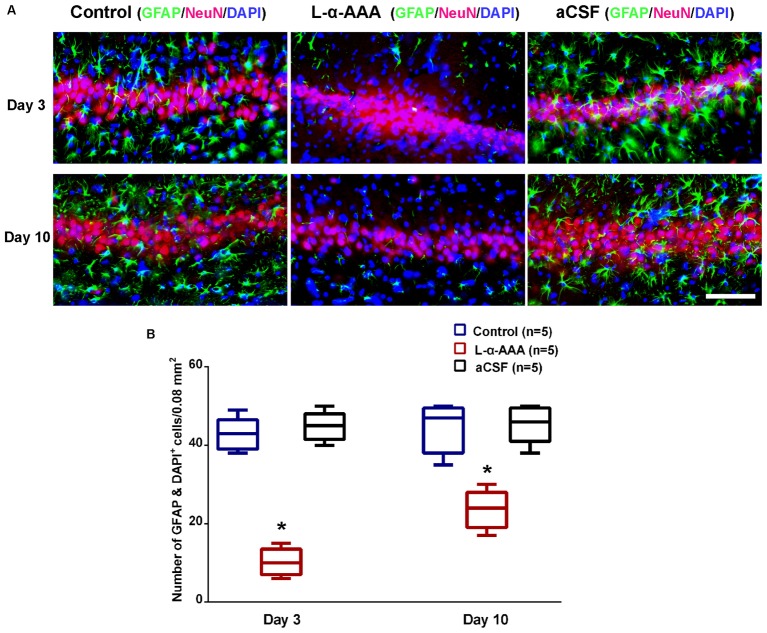
L-α-AAA injection induced the astrocytic depletion in hippocampal CA3. **(A)** Representative GFAP/NeuN/DAPI stained images of CA3 region after L-α-AAA or aCSF injection at days 3 and 10. L-α-AAA induced the depletion of astrocytes (GFAP&DAPI ^+^ cells) in CA3 region at day 3 and 10. **(B)** Number of astrocytes (GFAP&DAPI ^+^ cells) was calculated in a 0.08 mm^2^ area (up to 100 μm above and below the CA3 nerve cell layer, 400 μm width to the left and right) using NDP.view 2.0 software. A remarkable decrease in astrocytes number is seen in the L-α-AAA-injected group compared with the control group (i.e., the CA3 region contralateral to the L-α-AAA-injected region) and the aCSF-injected group at days 3 and 10. ^∗^*p* < 0.01. Scale bar = 100 μm.

To evaluate whether astrocytes participate in neuroprotection in the hippocampal CA3 region, a model with decreased astrocyte numbers in CA3 was created by local injection of L-α-AAA. To confirm decreased astrocyte numbers after L-α-AAA treatment, immunofluorescence staining for GFAP, NeuN, and DAPI staining were performed at day 3 and 10 after the injection, and the number of astrocytes (GFAP&DAPI ^+^ cells) in the hippocampal CA3 region was calculated. We found a significant decrease in GFAP expression in the L-α-AAA-injected group compared with the control group (CA3 region contralateral to the L-α-AAA-injected side) and the aCSF-injected group (**Figure 3**). We calculated the number of GFAP&DAPI ^+^ cells in a 0.08 mm^2^ area (up to 100 μm above and below the CA3 nerve cell layer, 400 μm width to the left and right) using NDP.view 2.0 software (**Figure 3A**). The number of GFAP&DAPI ^+^ astrocytes was as follows, for day 3 and 10, respectively (mean ± SD): aCSF-injected group, 44.8 ± 3.7 and 45.4 ± 4.8; control group, 42.8 ± 4.2, and 44.4 ± 6.3; L-α-AAA-injected group, 10.2 ± 3.5 and 23.6 ± 4.9. There was no significant difference between the aCSF-treated group and the control group. However, we found a significant difference between the L-α-AAA group and the other two groups at all time points (*p* < 0.01) (**Figure 3B**). The number of GFAP&DAPI ^+^ astrocytes was found to be significantly decreased in all L-α-AAA-injected sub groups (i.e., days 3 and 10). Astrocyte numbers were decreased by L-α-AAA for at least 10 days. These results establish our model of decreased astrocyte numbers in the hippocampal CA3 region.

### L-α-AAA Injection Does Not Change the rCBF Pattern Upon 4-Vessel Occlusion

**FIGURE 4:**
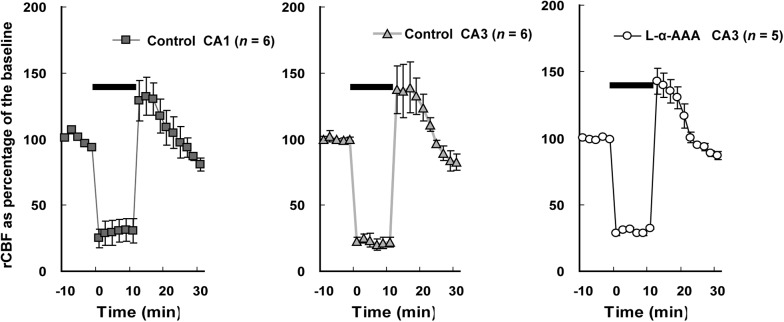
Regional cerebral blood flow (rCBF) changes during transient forebrain ischemia in the hippocampal CA1 and CA3 regions. The initial rCBF of each brain was set as the baseline, and percentage of the baseline was plotted. Injection of L-α-AAA does not change the rCBF pattern. Values are means ± SD. The horizontal bar indicates the duration of ischemia.

The rCBF in the hippocampal CA1 and CA3 regions was measured by LDF during 4-vessel occlusion (**Figure 4**). In the control group, in the CA1 and CA3 regions, the rCBF declined to 25–30% of baseline during the period of 4-vessel occlusion. At the beginning of reperfusion, we observed a transient overshoot followed by a gradual decline to the baseline level over a few minutes. In the L-α-AAA-injected group, the rCBF pattern was similar to that of the control group. As a result, we found that the patterns of rCBF change in hippocampal CA1 and CA3 were the same and the injection of L-α-AAA did not change the rCBF pattern elicited by forebrain ischemia/reperfusion.

### Loss of Astrocytes Induced Delayed Neuronal Death in the Hippocampal CA3 Region After Transient Forebrain Ischemia

**FIGURE 5:**
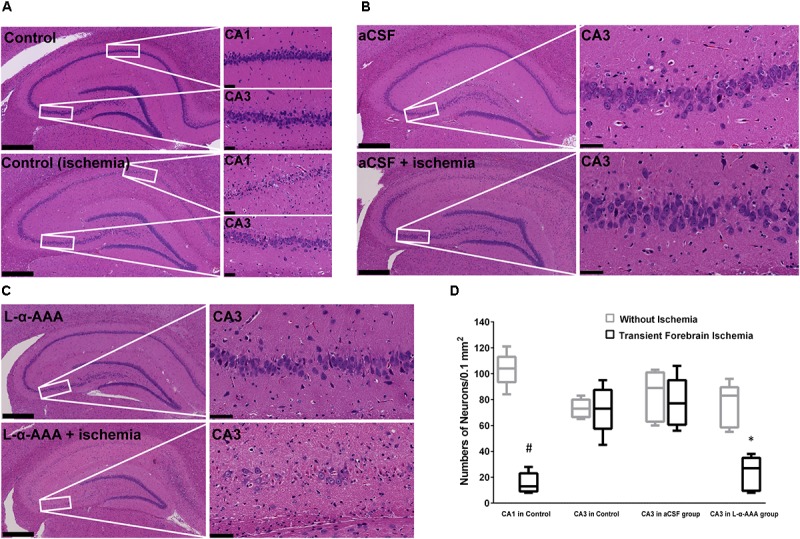
Representative hematoxylin-eosin-stained images of brain slices showing that L-α-AAA induced delayed neuronal death in the hippocampal CA3 region 7 days after transient forebrain ischemia. **(A)** Control groups: delayed neuronal death is found in the CA1 region after ischemia, but neurons in CA3 are spared. **(B)** aCSF-treated group: sham injection does not induce delayed neuronal death in the CA3 region in either the ischemic or the non-ischemic groups. **(C)** L-α-AAA group: L-α-AAA induces delayed neuronal death in the hippocampal CA3 region 7 days after transient forebrain ischemia, while neuronal death is not observed in the non-ischemic group. Scale bars on the left sides of panels = 500 μm. Scale bars on the right sides of panels = 50 μm. **(D)** Shown are numbers of pyramidal neurons per 0.1 mm^2^ in the CA1 and/or CA3 region (up to 100 μm above and below the CA3 nerve cell layer, 500 μm width to the left and right, magnified areas in **A**–**C**). *n* = 5 in each group. ^#^*p* < 0.01 compared with no-ischemia control of CA1. ^∗^*p* < 0.01 compared with controls, aCSF groups and no-ischemia L-α-AAA group of CA3.

We performed HE staining to investigate the response of neurons to transient forebrain ischemia in L-α-AAA-treated, aCSF-treated, and control groups (**Figure 5**). In the latter two groups, we observed delayed neuronal death in hippocampal CA1 region 7 days after transient forebrain ischemia but neurons in the CA3 region were spared (**Figure 5A**), a phenomenon which has been extensively reported in previous studies (Kirino et al., 1984). Interestingly, in the L-α-AAA-treated + transient forebrain ischemia group, delayed neuronal death was also observed in the hippocampal CA3 region 7 days after ischemia (**Figure 5C**). Delayed neuronal death was not observed in CA3 regions receiving L-α-AAA treatment without ischemia. Thus, L-α-AAA did not directly cause the neuronal death observed in the hippocampal CA3. Delayed neuronal death was not observed in the CA3 region of the sham-treated group (i.e., receiving ischemia without L-α-AAA treatment) (**Figure 5B**). The number of neurons/0.1 mm^2^ (up to 100 μm above and below the CA3 nerve cell layer, 500 μm width to the left and right) at day 10 was as follows, with and without ischemia, respectively (mean ± SD): CA1 in control group 15.4 ± 7.1 and 103.4 ± 11.7; CA3 in control group 72.6 ± 16.3 and 73.2 ± 6.4; CA3 in aCSF group 77.6 ± 17.2 and 83.4 ± 17.4; CA3 in L-α-AAA group, 23.2 ± 11.8, and 75.8 ± 15.1. In the L-α-AAA + ischemia group, we found a significant decrease in the number of neurons in the hippocampal CA3 compared with control and aCSF-injected groups (*p* < 0.01) (**Figure 5D**).

### In Transient Forebrain Ischemia/Reperfusion, Loss of Astrocytes Induces Intracellular Calcium Responses in the CA3 Region Similar to Those in CA1

**FIGURE 6:**
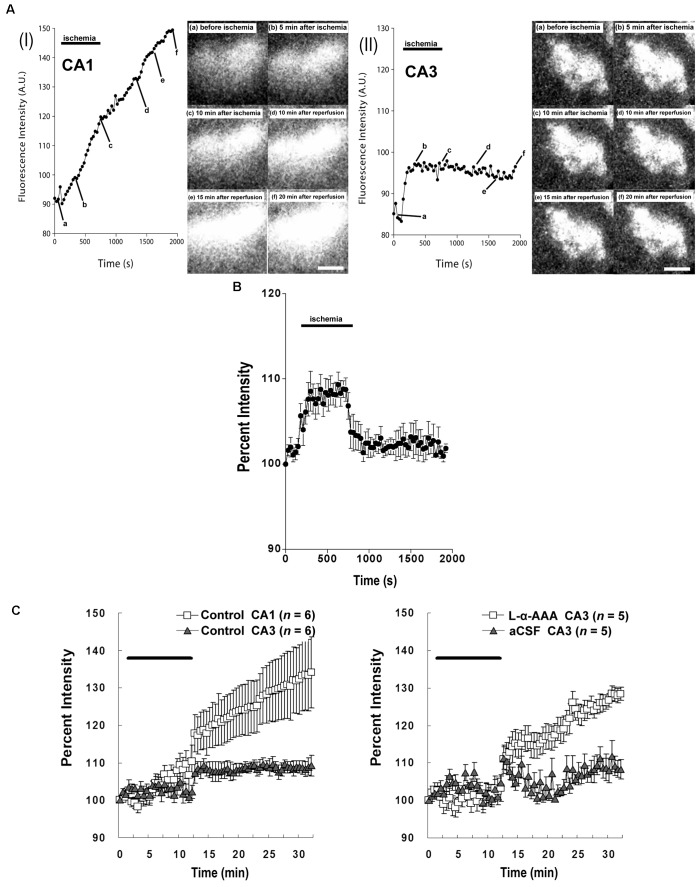
Intracellular Ca^2+^ fluorescence intensity in the hippocampal CA1 and CA3 regions following transient forebrain ischemia. Representative fluorescence-intensity images of intracellular Ca^2+^ at different time points in the CA1 **(AI)** and CA3 regions **(AII)** of the control group. **(B)** Changes in the apparent fluorescence intensity of fluoromicrospheres during ischemia/reperfusion (*n* = 6). **(C)** Intracellular Ca^2+^ % fluorescence intensity corrected with data obtained from the fluoromicrospheres. Comparing fluorescence intensity in the control group between CA1 and CA3 (Left) and comparing the L-α-AAA-treated group with the aCSF-treated group, both in CA3 (Right), L-α-AAA appears to induce calcium responses in hippocampal CA3 neurons following transient forebrain ischemia/reperfusion very similar to those normally seen in the CA1 region. Values are expressed as means ± SD. The black, horizontal bar indicates the duration of ischemia. White scale bars = 100 μm.

Our study confirmed that the intracellular Ca^2+^ responses after transient forebrain ischemia in hippocampal CA1 and CA3 regions’ cells are significantly different. In our imaging observations, transient forebrain ischemia causes a persistent increasing trend in [Ca^2+^]_i_ in the hippocampal CA1 region after ischemia/reperfusion (**Figure 6AI**). On the other hand, [Ca^2+^]_i_ in the CA3 region did not persistently trend upward, remaining constant at a somewhat elevated level (**Figure 6AII**).

As we show in **Figure 4**, 4-vessel occlusion induced a decrease in rCBF. Because of this change, the apparent fluorescence intensity of the fluoromicrospheres in the hippocampal CA3 region increased during the 10-min ischemic period and decreased immediately to baseline after reperfusion (**Figure 6B**). The ischemia/reperfusion procedure would have affected the fluo3/AM fluorescence intensity in the CA3 region in exactly the same way.

The corrected Ca^2+^ fluorescence intensity (% of baseline) is shown in **Figure 6C**. This figure quantitatively aggregates results across subjects. Significantly, we observed a different [Ca^2+^]_i_ pattern from that of the control group or the aCSF-injected group in the hippocampal CA3 region. Astrocyte damage in the hippocampal CA3 caused a continuous increase in the Ca^2+^ fluorescence intensity after brain ischemia/reperfusion, which was comparable to that in the hippocampal CA1 region of the control group. A sham-treated group (aCSF injected into CA3) did not have the increased Ca^2+^ fluorescence intensity in CA3, its [Ca^2+^]_i_ response being very similar to that of the CA3 of the control group.

## Discussion

In the present study, we observed that: (1) loss of astrocytes induced delayed neuronal death in the hippocampal CA3 region following transient forebrain ischemia; (2) loss of astrocytes did not affect the rCBF response to transient forebrain ischemia; and (3) loss of astrocytes produced intracellular calcium responses to transient forebrain ischemia/reperfusion in the CA3 region similar to those seen in the CA1 region. The results clearly show that astrocytes protect the hippocampal CA3 pyramidal neurons from delayed neuronal death following insults such as transient forebrain ischemia.

In agreement with previous reports (Pulsinelli and Brierley, 1979; Pulsinelli and Buchan, 1988), we observed delayed neuronal death in hippocampal CA1 neurons but not CA3 neurons in our controls. However, we observed delayed neuronal death in hippocampal CA3 neurons 7 days after transient forebrain ischemia after the astrocytes had been damaged locally by L-α-AAA injection. Considering that the injection of L-α-AAA may have influenced the rCBF and that operations may induce neuronal death, the rCBF was measured and a sham- (aCSF-) treated group was set up. According to our results, the injection of L-α-AAA *per se* did not change the rCBF pattern and the aCSF injection operation *per se* did not induce delayed neuronal death (**Figures 4**, **5B**). Moreover, L-α-AAA injection alone (i.e., without ischemia/reperfusion) did not cause delayed neuronal death (**Figure 5C**). Delayed neuronal death developed in the hippocampal CA3 after transient forebrain ischemia only when the local astrocytes had been damaged. Therefore, our results indicate that the loss of astrocytes in the CA3 region led to the observed delayed neuronal death of CA3 pyramidal neurons after transient forebrain ischemia. In other words, astrocytes play a neuroprotective role in the hippocampal CA3 region against brain ischemia.

Consistent with our results, Ouyang et al. (2007) reported that selective dysfunction of hippocampal CA1 astrocytes contributes to delayed neuronal damage after transient forebrain ischemia. However, these authors focused on the loss of glutamate transporter-1 function in CA1 astrocytes. While several reports suggested possible mechanisms underlying the neuroprotection conferred by astrocytes, the actual mechanisms remained unknown until now. Astrocytes are known to protect neurons from the insults of oxidative stress and excitotoxicity after brain ischemia (Rossi et al., 2007; Takano et al., 2009; Zhao and Rempe, 2010). Astrocytic brain-derived neurotrophic factor is known to reduce neuronal apoptosis in the ischemic boundary zone and improve functional recovery (Qu et al., 2010). Astrocytes are also known to play neuroprotective roles during brain ischemia by the expression of astrocyte-enriched miRNAs, including miR-181, miR-29 families, and miR-146a (Ouyang et al., 2014). The astrocytic HMGB1/IL-6 signaling pathway participates in angiogenesis, neurogenesis, and the promotion of post-stroke functional recovery (Chen et al., 2017). Astrocytes can upregulate pentraxin 3 to maintain blood-brain-barrier integrity by regulating vascular endothelial growth factor-related mechanisms (Shindo et al., 2016). Astrocytes can also mobilize functional mitochondria and transport them to neurons after stroke to contribute to the neuroprotection and functional recovery of neurons (Hayakawa et al., 2016).

Another important finding of our study was that the intracellular Ca^2+^ responses in CA1 and CA3 regions after transient forebrain ischemia were significantly different (**Figure 6**). While the [Ca^2+^]_i_ in hippocampal CA1 persistently trended upward after transient forebrain ischemia/reperfusion, this effect was not seen in the hippocampal CA3. Furthermore, delayed neuronal death occurs in the hippocampal CA1 region after transient forebrain ischemia, but not in the CA3 region (Kirino et al., 1984). This contrast in patterns of [Ca^2+^]_i_ increase following ischemia may be one of the reasons for the selective vulnerability of hippocampal CA1 neurons to ischemia. The differences between the hippocampal CA1 and CA3/dentate gyrus (DG) regions have been extensively studied. For instance, the expression of glutamate transporter-1 and heat shock protein 70 in the CA1 region are higher than in the CA3/DG region; a strongly activated, extracellular signal-regulated kinase 5 was seen in the CA3/DG region but not in the CA1 following transient forebrain ischemia; the mitochondria isolated from CA1 neurons were found to be more sensitive to calcium-induced swelling than those from the CA3 region (Kawagoe et al., 1993; Mattiasson et al., 2003; Wang et al., 2005; Zhang et al., 2011). Our study describes a novel, further difference between hippocampal CA1 and CA3 region *in situ*, which may lead to the discovery of the mechanism of selective, delayed neuronal damage in hippocampal CA1 neurons.

In the present study, astrocytes in the hippocampal CA3 region were experimentally damaged using L-α-AAA. We then found that the intracellular Ca^2+^ fluorescence intensity of hippocampal CA3 region increased continuously post-ischemia, which is very similar to the pattern in the hippocampal CA1 region (**Figure 6**). We concluded that the loss of astrocytes caused intracellular Ca^2+^ overloading in CA3 region’s cells, including neurons, and other cells, after transient forebrain ischemia/reperfusion, eventually leading to neuron death. Intracellular-Ca^2+^ overload is one of the most important mechanisms of neuronal death following brain ischemia (Deb et al., 2010; Szydlowska and Tymianski, 2010). According to our results, the astrocytes may play a neuroprotective role in the hippocampal CA3 region after transient forebrain ischemia by suppressing the intracellular Ca^2+^ overload of CA3 cells, including neurons. However, the mechanism of this phenomenon is still unclear. Possible explanations are as follows: (1) excitatory neurotransmitters accumulate in the extracellular space and synaptic cleft and over-stimulate several receptors including the *N*-methyl-*D*-aspartate-preferring (NMDAR) type and the α-amino-3-hydroxy-5-methyl-4-isoxazolepropionic acid-preferring (AMPAR) type of glutamate receptor; (2) this process induces a large influx of Ca^2+^ into neurons and results in Ca^2+^ cytotoxicity; (3) astrocytes might remove the excess glutamate from the synaptic cleft to limit the overload of intracellular Ca^2+^ in neurons (Deb et al., 2010; Szydlowska and Tymianski, 2010; Becerra-Calixto and Cardona-Gómez, 2017). The hippocampal CA3 astrocytes may have a special, enhanced ability to regulate glutamate and Ca^2+^ toxicity. The differences between hippocampal CA1 and CA3 astrocytes mentioned above may contribute to the capacity for suppressing intracellular Ca^2+^ overloading after ischemia/reperfusion in the hippocampal CA3 region cells.

## Conclusion

In summary, our study observed different [Ca^2+^]_i_ changes in hippocampal CA1 and CA3 regions after transient forebrain ischemia, and astrocytes were found to protect neurons against transient forebrain ischemia by suppressing the intracellular Ca^2+^ overload of CA3 region’s cells, including neurons. This novel finding reveals a new approach to explaining the selective vulnerability of hippocampal CA1 neurons and the neuroprotective role of astrocytes after transient forebrain ischemia. Furthermore, our study will most likely provide a new therapeutic strategy for brain ischemic diseases, targeted to astrocytes.

## Author Contributions

SY conceived and designed the experiments. CS and YW performed the experiments. SY and CS interpreted the data and prepared the figure. CS, YF, and SY wrote and revised the manuscript.

## Conflict of Interest Statement

The authors declare that the research was conducted in the absence of any commercial or financial relationships that could be construed as a potential conflict of interest.
